# Neural cell adhesion molecule *Negr1* deficiency in mouse results in structural brain endophenotypes and behavioral deviations related to psychiatric disorders

**DOI:** 10.1038/s41598-019-41991-8

**Published:** 2019-04-01

**Authors:** Katyayani Singh, Mohan Jayaram, Maria Kaare, Este Leidmaa, Toomas Jagomäe, Indrek Heinla, Miriam A. Hickey, Allen Kaasik, Michael K. Schäfer, Jürgen Innos, Kersti Lilleväli, Mari-Anne Philips, Eero Vasar

**Affiliations:** 1 Department of Physiology, Institute of Biomedicine and Translational Medicine, University of Tartu, 19 Ravila Street, 50411 Tartu, Estonia; 2 Centre of Excellence in Genomics and Translational Medicine, University of Tartu, 19 Ravila Street, 50411 Tartu, Estonia; 3 Institute of Molecular Psychiatry, University of Bonn, Sigmund-Freud-Str.25, 53127 Bonn, Germany; 4 Department of Psychology, UiT The Arctic University of Norway, Postboks 6050 Langnes, 9037 Tromso, Norway; 5 Department of Pharmacology, Institute of Biomedicine and Translational Medicine, University of Tartu, 19 Ravila Street, 50411 Tartu, Estonia; 6 Department for Anesthesiology, University Medical Center and Focus Program Translational Neuroscience (FTN), Johannes Gutenberg-University Mainz, Mainz, Germany

## Abstract

Neuronal growth regulator 1 (NEGR1) belongs to the immunoglobulin (IgLON) superfamily of cell adhesion molecules involved in cortical layering. Recent functional and genomic studies implicate the role of NEGR1 in a wide spectrum of psychiatric disorders, such as major depression, schizophrenia and autism. Here, we investigated the impact of *Negr1* deficiency on brain morphology, neuronal properties and social behavior of mice. *In situ* hybridization shows *Negr1* expression in the brain nuclei which are central modulators of cortical-subcortical connectivity such as the island of Calleja and the reticular nucleus of thalamus. Brain morphological analysis revealed neuroanatomical abnormalities in *Negr1*^*−/−*^ mice, including enlargement of ventricles and decrease in the volume of the whole brain, corpus callosum, globus pallidus and hippocampus. Furthermore, decreased number of parvalbumin-positive inhibitory interneurons was evident in *Negr1*^*−/−*^ hippocampi. Behaviorally, *Negr1*^*−/−*^ mice displayed hyperactivity in social interactions and impairments in social hierarchy. Finally, *Negr1* deficiency resulted in disrupted neurite sprouting during neuritogenesis. Our results provide evidence that NEGR1 is required for balancing the ratio of excitatory/inhibitory neurons and proper formation of brain structures, which is prerequisite for adaptive behavioral profiles. Therefore, *Negr1*^*−/−*^ mice have a high potential to provide new insights into the neural mechanisms of neuropsychiatric disorders.

## Introduction

Nosologically distinct psychiatric disorders such as schizophrenia (SCZ), major depressive disorder (MDD), bipolar disorder (BP), autism spectrum disorders (ASD), and attention-deficit hyperactivity disorder (ADHD) share a common genetic etiology with a diverse set of partially overlapping symptoms^1^. Converging evidence suggests the highly heritable and shared polygenic traits, that contribute to the abnormalities in neural connectivity overlap across disorders^1–3^.

Impaired cortical-subcortical integrity has been involved in the development of psychiatric disorders like SCZ^4^, MDD^5^, ASD^6^, BP^7^ and in the etiology of psychological and cognitive symptoms in neurodegenerative disorders like Alzheimer’s disease (AD)^8^ and Parkinson’s disease (PD)^9^. Proper connectivity of brain structures is essential for the functioning of cortical-subcortical interactions such as cortico-striatal circuits, prefrontal-amygdala circuits, prefrontal-hippocampal and thalamo-cortical circuitry. Neuroimaging studies also indicate common cross-disorder volumetric alterations of cortical and subcortical brain regions, the most replicated of these being the enlargement of ventricles, and the reduced volume of the hippocampus, frontal cortex and corpus callosum^10–13^.

Neuronal growth regulator 1 (NEGR1) is a member of the IgLON superfamily of cell adhesion molecules (CAMs), which also include limbic system associated membrane protein (Lsamp), neurotrimin (Ntm), opioid-binding protein/cell adhesion molecule like (Opcml) and IgLON-5^14^. Accumulating evidence suggests the involvement of NEGR1 in a wide spectrum of psychiatric conditions. NEGR1 is expressed in neuronal somata and dendritic synaptic vesicles of various brain regions in the developing and adult brain, suggesting its function in brain connectivity^15–17^. Large-scale genome-wide association studies (GWAS) indicate polymorphisms present in the *NEGR1* gene to be associated with the risk for SCZ^18^, MDD^19^ and AD^20^. Variations in *NEGR1* are linked with human intelligence^21^ and dyslexia^22^. Polymorphisms in *NEGR1* have also been implicated in low white matter integrity, which could be the underlying risk factor for many psychiatric phenotypes^23^. Two siblings with a microdeletion in chromosome 1p31.1, including partial deletion of the *NEGR1* gene have been reported to have neuropsychiatric, behavioral and learning difficulties^24^. Additional rare deletional cases associated with *NEGR1* in patients cause intellectual disability and severe language impairment^25^.

The levels of NEGR1 protein and transcripts are elevated in the post-mortem prefrontal cortex (PFC)^26^ and dorsolateral prefrontal cortex (DLPFC)^27^ of schizophrenic patients. In addition, increased level of *NEGR1* transcripts has been reported in the DLPFC of patients with MDD in comparison with healthy controls^28^. NEGR1 is among the biomarkers which have been picked up in the cerebrospinal fluid proteome signatures in MDD and BP exclusive of SCZ^29^. Another study showed increased NEGR1 levels in human cell lines which are treated with clozapine, suggesting NEGR1 as a target of antipsychotic drugs^30^. In treatment of Dark Agouti rats with common antidepressant venlafaxine, a serotonin and noradrenaline reuptake inhibitor, upregulation of NEGR1 has been observed as a response^31^. Specific variants in *NEGR1* gene locus have been implicated in human obesity, body mass index^32,33^ and psychological traits commonly linked with eating disorders^34^. The body mass phenotype could be related to the interaction of NEGR1 with the Niemann-Pick disease Type C2 (NPC2) protein that alters cholesterol transport^35^.

Evidence from high-throughput single cell transcriptomics (RNA seq) study on mouse brain cell types showed Negr1 expression in neurons, astrocytes, oligodendrocyte progenitors cells, newly formed oligodendrocytes and (Tmem119+) microglia at P7^36^. Functional studies in cultured cells have shown that NEGR1 can regulate neuronal outgrowth, arborisation and synaptogenesis by creating a permissive substrate during the development of cortical and hippocampal neurons via the influence of FGFR2 signalling pathway^37–39^. NEGR1 also functions as a trans-neural growth-promoting factor for outgrowing axons following hippocampal denervation^37^. Our study using *Negr1*^*−/−*^ mice provides evidence that *Negr1* is related to neuronal connectivity and behavior. *Negr1*^*−/−*^ mice exhibit altered entorhinal fibre projections and neurotransmitter receptor ligand binding in distinct hippocampal subfields. Behavioral deficits in *Negr1*^*−/−*^ mice include increased seizure susceptibility, impaired social approach and learning deficits^40^. A recent study has demonstrated that NEGR1 and FGFR2 interactions are required for neuronal migration during cortical development^41^. Impaired ERK and AKT signalling were involved in the core behavioral alterations related to ASD in juvenile *Negr1*^*−/−*^ mice.

Our aim was to characterise the alterations in the brain of *Negr1* deficient mice from early neuritogenesis to the anatomy of brain structures to better understanding the changes in the neuronal substrate underlying behavioral deviations in this mouse model.

## Results

### Altered brain anatomy in *Negr1*^*−/−*^ mice

**Figure 1:**
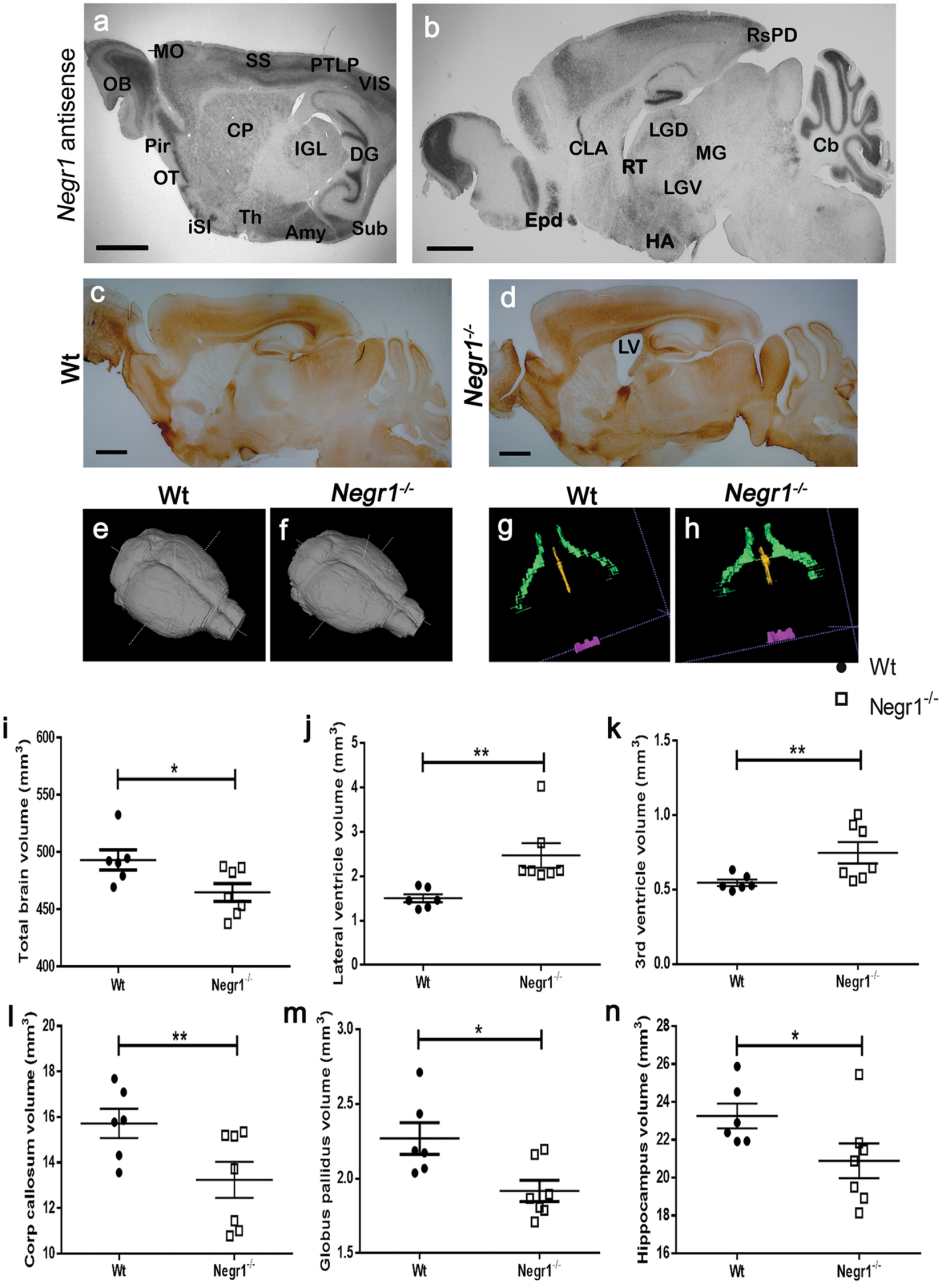
Neuroanatomy of *Negr1* in adult mouse brain. Expression of *Negr1* by *in situ* hybridisation in adult sagittal brain sections **(a**,**b)**. *OB* olfactory bulb, *Pir* piriform cortex, *OT* olfactory tubercle, *iSI* island of Calleja, *Th* thalamus, *Amy* amygdala, *Sub* subiculum, *DG* dentate gyrus, *IGL* inter geniculate lateral geniculate complex, *CP* caudate putamen, *MO* somatomotor cortex, *SS* somatosensory cortex, *PTLP* posterior parietal association area, *VIS* visual area, *Epd* endopiriform cortex, *HA* hypothalamus, *Cb* cerebellum, *RsPD* retrosplenial cortex, *CLA* claustrum, *RT* reticular nucleus of thalamus, *LGD/V* dorsal/ventral part of lateral geniculate complex, *MG* medial geniculate nucleus. Gross cytoarchitecture shown by neurofilament immunostaining in Wt **(c)**, enlarged ventricles in *Negr1*^*−/−*^ mice brain (**d**), *LV* lateral ventricles. A 3D reconstruction of the Wt (**e**) and *Negr1*^*−/−*^ (**f**) mice brains and ventricles **(**green: lateral ventricles, yellow: third ventricle and purple: fourth ventricle) in Wt (**g**) and *Negr1*^*−/−*^ (**h**) brains. Ventricle enlargement is observed in the *Negr1*^*−/−*^ *mice*. Quantitative analysis of effects of Negr1 deletion in mice on the volume of total brain (**i**), lateral ventricles (**j**), third ventricles (**k**), corpus callosum **(l)**, globus pallidus **(m)** and in hippocampus (**n**). Data represent mean ± SEM, *p < 0.05, **p < 0.01, ***p < 0.001, Mann-Whitney *U* test (Wilcoxon rank sum test).

First, *in situ* hybridisation was carried out to label the *Negr1* expression in adult mouse brain. *Negr1* is expressed extensively in the forebrain and cerebellum. Strong expression was observed in all cortical layers in different areas (somatomotor, somatosensory, parietal association area, visual area, retrosplenial area), in the limbic system (hippocampus:DG, CA1-3 subfields), entorhinal cortex, subiculum, amygdala, hypothalamus, islands of Calleja, olfactory bulb, olfactory tubercle, lateral geniculate complex and reticular nuclei of thalamus), globus pallidus, and granular layer of cerebellum along with caudate putamen (Fig. 1a,b). No *Negr1* signal was detected in *Negr1*^*−/−*^ brain sections (Supplementary Fig. S1). For the initial screening of sub-regional organisation in *Negr1*^*−/−*^ mouse brain we carried out an immunostaining detecting neurofilament. There were no obvious changes in the gross anatomy of *Negr1*^*−/−*^ brain except for remarkably enlarged lateral ventricles were observed. Cytoarchitecture and the fibre-tracts are illustrated in Fig. 1c,d.

To study neuroanatomical changes, we analysed the volume of the whole brain and selected brain regions in *Negr1*^*−/−*^ mice compared to their Wt littermates using high resolution MRI. No significant differences in body weight and brain weight of *Negr1*^*−/−*^ mice were observed compared to their Wt littermates (Table 1). The analysed brain structures, including the whole brain, ventricular system, white matter tracts, and cortical and subcortical grey matter structures were depicted in Table 1. We detected a small but significant (5.7%) reduction in total brain volume in *Negr1*^*−/−*^ mice as compared to Wt controls (Fig. 1e,f,i). Also, *Negr1*^*−/−*^ mice had significantly enlarged lateral ventricles (64.6%), third ventricle (37%) and fourth ventricle (35.7%) (Fig. 1g,h,j,k). Regarding white matter areas, the corpus callosum was found to be significantly reduced (15.7%) (Fig. 1l), while other white matter tracts like the anterior commissure (anterior and posterior), internal capsule, fornix and fimbria remained unchanged. Enlargement of the ventricular system in *Negr1*^*−/−*^ mice might partly reflect the reduction in the volume of some cortical and subcortical areas. We found a significant reduction in the size of the globus pallidus (15.5%) and hippocampus (10%) (Fig. 1m,n). No changes were observed in the frontal cortex, olfactory system (olfactory bulb, olfactory tubercle, lateral olfactory tract and rhinocele), striatum, hypothalamus, medulla, midbrain, brain stem and cerebellum volume (Table 1).

**Table 1:** Weight (g) and volume (mm^3^) measurements (mean ± SEM) of selected regions in the *Negr1*^−/−^ compared to the corresponding Wt. *P* values as determined by Mann-Whitney U test (Wilcoxon rank sum test). (Bold *p*-value means significant difference as *p < 0.05; **p < 0.01, ***p < 0.001).

	Wt	*Negr1* ^−/−^	% Diff	*P*-value
Body weight (g)	32.7 ± 3.6	31.2 ± 5	−4.7	0.32
Brain weight	0.45 ± 0.022	0.45 ± 0.028	−0.6	0.88
Total brain volume (mm^3^)	493.2 ± 21.5	465 ± 20.4	−5.7	**0.03**
**White matter regions (mm** ^3^ **)**				
Corpus callosum	15.71 ± 1.5	13.24 ± 2.1	−15.7	**0.04**
Ant commissure (anterior)	1.4 ± 0.15	1.3 ± 0.2	−8.9	0.31
Ant commissure (posterior)	0.35 ± 0.9	0.36 ± 0.06	1.9	0.77
Internal capsule	2.1 ± 0.5	1.9 ± 0.56	−10.18	0.31
Fornix	0.49 ± 0.07	0.45 ± 0.06	−7.83	0.47
Fimbria	1.8 ± 0.33	1.7 ± 0.21	−4.7	0.47
**Ventricles (mm** ^3^ **)**				
Lateral Ventricle	1.5 ± 0.22	2.47 ± 0.73	64.6	**0.002**
3^rd^ Ventricle	0.54 ± 0.05	0.74 ± 0.18	37	**0.02**
4^th^ Ventricle	0.14 ± 0.02	0.19 ± 0.06	35.7	0.06
**Cortical and sub-cortical grey matter regions (mm** ^3^ **)**				
Olfactory system	33.5 ± 2.9	32.9 ± 2.1	−1.9	0.66
Frontal cortex	52.6 ± 6.5	48.3 ± 2.8	−8.2	0.11
Striatum	18.5 ± 1.2	17.7 ± 1.2	−4.2	0.19
Globus pallidus	2.2 ± 0.5	1.9 ± 0.18	−15.5	**0.03**
Hippocampus	23.2 ± 1.6	20.8 ± 2.4	−10	**0.02**
Thalamus	15.2 ± 0.9	15.1 ± 1.5	−1.04	0.77
Hypothalamus	10.3 ± 0.7	9.9 ± 0.6	−4.21	0.47
Midbrain	8.8 ± 0.5	9.0 ± 1.2	2.03	0.66
Pons	16.7 ± 1.8	15.3 ± 1.5	−8.3	0.31
Medulla	21.0 ± 3.2	22.7 ± 3.7	8.16	0.39
Cerebellum	55.3 ± 1.9	57.5 ± 2.1	3.99	0.19

### Impact of Negr1 on hippocampal neuronal population

**Figure 2:**
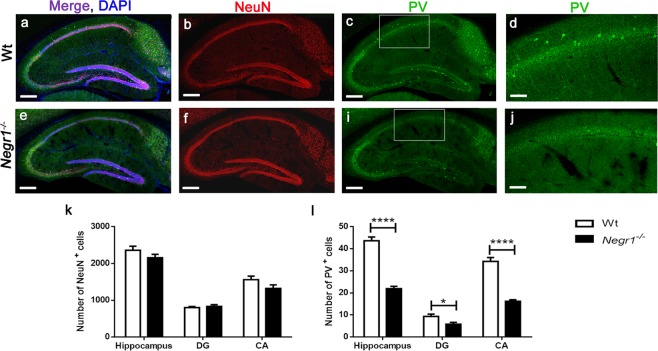
Reduced number of parvalbumin (PV) positive interneurons in the hippocampus of *Negr1*^*−/−*^ mice. Representative confocal images of the sagittal section of brains of Wt **(a–d**) and *Negr1*^*−/−*^ **(e–j)** mice for NeuN (red), PV (green) and DAPI (blue) immunostaining. Scale bar is 250 μm for a**–**c, e**–**g and 100 μm for d and j. Graph represents mean number of NeuN positive cells **(k**) and PV positive cells (**l**) in total hippocampus, DG and in CA region. N = 3 mice for both genotype with 8–9 sections per brain. Data represent mean ± SEM, *p < 0.05, **p < 0.01, ***p < 0.001, Mann–Whitney *U* test.

Since Negr1 is expressed in hippocampus and we have observed reduced hippocampal volume in *Negr1*^*−/−*^ mice, we examined whether Negr1 deficiency affect the whole neuronal population in hippocampus or some specific neuronal subtypes. We found no significant difference in number of NeuN (pan-neuronal marker) positive neurons (total hippocampal region, p = 0.13; dentate gyrus, DG = 0.40; Cornu Ammonis, CA = 0.16) (Fig. 2a,b,e,f,k). However, the number of parvalbumin (PV) positive interneurons was found to be significantly reduced (p < 0.0001), in the CA (p < 0.0001) and in DG (p < 0.02) regions of the hippocampus (Fig. 2c,d,i,j,l,).

### Lack of barbering behavior and selective deficits in social interaction in *Negr1*^*−/−*^ mice

**Figure 3:**
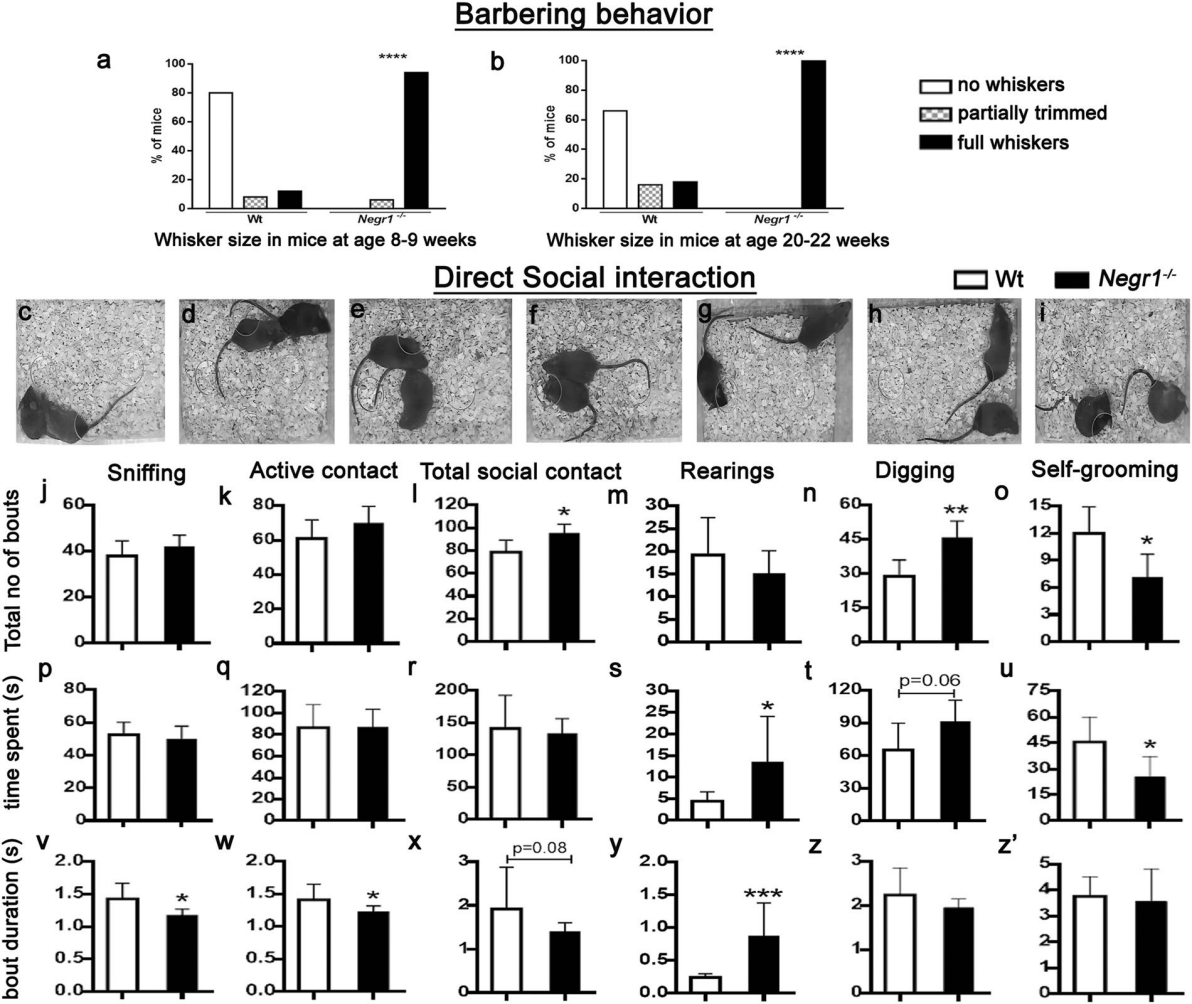
Barbering behavior and direct social interaction test. Percent of whisker size in mice at 8–9 weeks **(a)**, at 20–22 weeks **(b)** of age. Illustrative examples used for scoring direct social interactions such as sniffing other body parts **(c)**, anogenital sniffing **(d)**, active contact **(e)**, passive contact **(f)**, rearings **(g)**, digging **(h)** and self-grooming **(i)**. Graph represents total number of bouts **(j–o)**, time spent **(p–u)** and bout duration **(v–z’)** during each parameter of social interaction. Data represent mean ± SEM, *p < 0.05, **p < 0.01, ***p < 0.001, Mann-Whitney *U* test (Wilcoxon rank sum test).

After weaning, no whisker trimming or barbering behavior was observed in either genotype (the mice were group-housed by genotypes). At 8–9 weeks of age, most Wt mice were completely devoid of whiskers and had trimmed facial hair. In contrast to Wt littermates, all *Negr1*^*−/−*^ mice had full sets of whiskers and facial hair (χ^2^ = 143.72, p < 0.00001; Fig. 3a). Similar differences in barbering behavior were evident at 20–21 weeks of age (χ^2^ = 145.02, p < 0.00001; Fig. 3b). Notably, 100% of *Negr1*^*−/−*^ mice had a full set of whiskers and intact facial hair.

In our previous study, *Negr1*^*−/−*^ mice displayed impaired sociability and social dominance^40^. Here, we examined social interaction between two freely moving male mice (12–14 weeks old) of the same genotype as this test is considered to be more sensitive for studying social interactions in adult mice. During the 10 min direct social interaction test, aggressive behavior, anogenital sniffing, sniffing of other body parts, active contact, passive contact, rearings, digging and self-grooming were assessed (Fig. 3c–i). No attacks or aggressive behavior were registered during the interactions in either genotype. Mann-Whitney U test revealed that *Negr1*^*−/−*^ mice spent substantially less time in sniffing of other body parts than genitals (W = 82, p = 0.014) and in active contacts (W = 78, p = 0.035) and tended to have a shorter total social contact bout duration (W = 73, p = 0.08; Fig. 3j–l, p–r,v–x), whereas a higher number of bouts during total social contacts (W = 20.5, p = 0.027) was registered for *Negr1*^*−/−*^ mice as compared to Wt mice (Fig. 3l). Total social contact is a summarized measure reflecting the sum total of anogenital sniffing, sniffing of other body parts, passive contacts and active contacts (Table 2). As for non-social activities, *Negr1*^*−/−*^ mice spent more time on rearings (W = 18, p = 0.014) and had a longer rearing bout duration (W = 8, p = 0.0007); also, *Negr1*^*−/−*^ mice tended to spend more time digging (W = 25, p = 0.06) and had a larger number of digging bouts (W = 13, p = 0.0057) (Fig. 3m,n,s,t,y,z). In contrast, *Negr1*^*−/−*^ mice spent less time on self-grooming (W = 81, p = 0.019) and had less self-grooming bouts (W = 80.5, p = 0.02) (Fig. 3o,u, z’). Assessment of marble burying and tail-suspension tests revealed no differences between *Negr1*^*−/−*^ and Wt mice (Table 2).

**Table 2:** *Negr1*^−/−^ mice display alterations in social interactions and non-social interest. Social interaction scores during male-male direct interaction, marbles burying test (b) and tail suspension test (c). Data represent mean ± SEM and bold numbers represent significant differences, s (seconds), Mann–Whitney *U* test (Wilcoxon rank sum test).

Direct-Social Interaction test				
Parameter	Genotype	Total no of bouts	Time (s)	Bout duration (s)
Sum of total social contact	Wt	**73 ± 14.7**	134.8 ± 72	**2.2 ± 4.9**
	*Negr1* ^−/−^	**87.5 ± 11.5**	123.9 ± 32.9	**1.5 ± 2.9**
Active contact	Wt	56.8 ± 15.2	81.4 ± 30	**1.5 ± 2**
	*Negr1* ^−/−^	64.7 ± 14	81.1 ± 23.8	**1.3 ± 1.7**
Anogenital sniffing	Wt	23 ± 9.3	34 ± 22.2	1.3 ± 1.6
	*Negr1* ^−/−^	28 ± 9.2	37.3 ± 18.1	1.2 ± 0.3
Sniffing (other body parts)	Wt	38 ± 9	52.4 ± 10.7	**1.3 ± 0.3**
	*Negr1* ^−/−^	42 ± 7	49.5 ± 11.5	**1.1 ± 0.1**
Passive contact	Wt	18 ± 3.6	54.6 ± 83	2.8 ± 3.5
	*Negr1* ^−/−^	25 ± 12.4	46 ± 41	1.5 ± 0.6
Digging	Wt	**29 ± 9.9**	**65 ± 34.3**	2.1 ± 0.9
	*Negr1* ^−/−^	**46 ± 10.2**	**91 ± 27.6**	1.8 ± 0.3
Rearing	Wt	31 ± 10.9	5.9 ± 2.2	0.2 ± 0.1
	*Negr1* ^−/−^	25 ± 10.2	19.4 ± 16.9	0.7 ± 0.5
Self-grooming	Wt	**12 ± 4.1**	**45.6 ± 20**	3.5 ± 1
	*Negr1* ^−/−^	**7 ± 3.6**	**25.4 ± 16.5**	3.4 ± 1.8
Marble-burying test {Number of marble buried/displaced in 30 min}			Wt	6.15 ± 3.55
			*Negr1* ^−/−^	4.43 ± 2.6
Tail-suspension test {Immobility time duration (s)}			Wt	133 ± 25.4
			*Negr1* ^−/−^	118 ± 32.7

**Figure 4:**
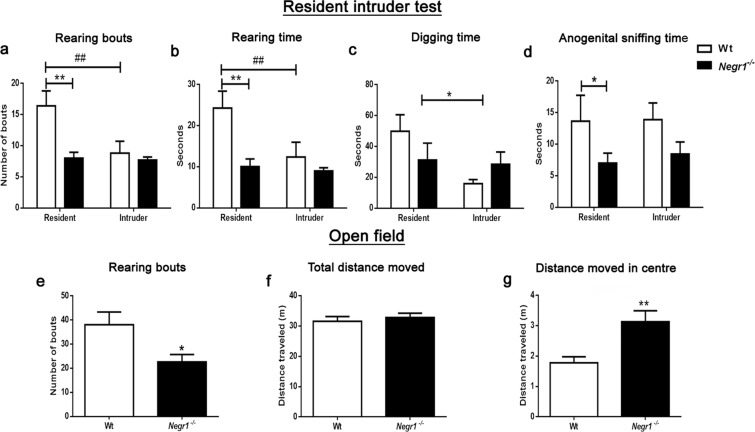
Resident intruder (RI) and Open field (OF) test. Graph represents total number of rearing bouts **(a)**, time spent in rearing **(b)**, digging **(c)** and in anogenital sniffing **(d)** during RIT. Total number of rearing bouts **(e)**, total distance travelled **(f)** and distance travelled in the center zone of the open field by 10 min period **(g)** during OFT. Data represent mean ± SEM, *p < 0.05, **; ^**##**^p < 0.01, by two-way ANOVA, followed by Newman-Keuls post hoc test (RI) and Mann–Whitney U test (OF).

Despite the fact that that *Negr1*^*−/−*^ mice showed no aggression, their social and non-social activities during the direct social interaction (DSI) test were nevertheless altered. We thus hypothesized that genotype-dependent differences in aggressive behavior could become evident in a social stress situation. We performed the resident-intruder (RI) test to assess aggressive behavior and social interaction. Neither Wt nor *Negr1*^*−/−*^ mice showed significant aggressive behavior, a surprising effect, possibly caused by the low propensity of the mice from the mixed (129S5/SvEvBrd × C57BL/6) genetic background to be aggressive. No main effects in sniffing were evident. The number of rearing bouts was significantly affected by genotype (F(1,36) = 8.64, p = 0.0057), resident/intruder group (F(1,36) = 5.97, p = 0.019) and genotype × group effect (F(1,36) = 5.10, p = 0.03) (Fig. 4a). Rearing time was significantly affected by genotype (F(1,36) = 9.18, p = 0.0045) and resident/intruder group (F(1,36) = 4.93, p = 0.033) (Fig. 4b). Digging time was significantly affected by resident/intruder group (F(1, 36) = 4.49, p = 0.04) (Fig. 4c). Anogenital sniffing time was significantly affected by genotype (F(1,36) = 4.88, p = 0.034) (Fig. 4d). Grooming bout duration was significantly affected resident/intruder group (F(1,36) = 9.90, p = 0.003). Different behavioral parameters analysed in RI test resulted in similar effects in both the resident and intruder *Negr1*^*−/−*^ mice attributing to the non-responsive phenotype of *Negr1*^*−/−*^ mice to social stressors (Fig. 4a–d). In the open field (OF) test the number of rearing bouts was reduced in *Negr1*^*−/−*^ mice (p = 0.049) (Fig. 4e). Total distance travelled was similar in both groups, whereas distance travelled in the centre was increased in *Negr1*^*−/−*^ mice compared with Wt mice (p = 0.0043) (Fig. 4f,g).

### Deficits in social behavior correlate with changes in the brain structure of *Negr1*^*−/−*^ mice

Next, we investigated the correlations between the regional volumetric changes in the brain and social interaction measures. Here, we also included sociability measurements from 3-chamber test which has been described in our previous study^40^. Interestingly, most of the significant correlations were found in *Negr1*^*−/−*^ mice: reduced total brain volume was negatively correlated with sniffing (bouts) and active contact (bouts and time), and positively correlated with passive and total social contact (length) (Table 3). Reduced hippocampal volume was positively correlated with passive contact (length) and the reduction in the volume of globus pallidus was negatively correlated with active contact (length) and positively with passive and total contact (length). Thinner corpus callosum was negatively correlated with sniffing (bouts), active contact (bouts and length) and 3-chamber sociability test. The size of the 3^rd^ ventricles was positively correlated with sniffing (bouts and length), active contact (bouts and length) and 3-chamber sociability test (Table 3). In Wt mice, non-social activity during direct social interactions showed positive correlations with the size of different brain regions, such as digging bouts with the volume of total brain and hippocampus. Rearing and self-grooming were positively correlated with the size of lateral and 3^rd^ ventricles (Supplementary Table S1). After pooling both genotypes, self-grooming was positively correlated with the total brain volume and hippocampal size, whereas self-grooming and rearings were negatively correlated with the volume of lateral ventricles (Supplementary Table S2).

**Table 3:** Correlations between the MRI indices and social indices of interest in *Negr1*^−/−^ mice. The behavioral measures have been presented in either bouts (-B), time (-T) or bout length (-L). Bold *p-*value represents a significant difference, *p < 0.05, **p < 0.01, ***p < 0.001 (Spearman’s rank-order correlation).

	Body wt	Brain wt	Total Brain	Hippo	CC	LV	3V	4V	GP
SNIF-B	−0.58	−0.16	−0.89**	−0.55	−0.98***	−0.25	0.87**	−0.44	−0.77
SNIF-T	−0.42	−0.06	−0.76	−0.71	−0.77	0.04	0.91**	−0.08	−0.77
SNIF-L	0.25	0.07	0.34	−0.17	0.49	0.59	−0.17	0.53	0.11
ACT-B	−0.61	−0.24	−0.92**	−0.59	−0.98***	−0.31	0.85**	−0.53	−0.78
ACT-T	−0.62	−0.32	−0.94*	−0.76	−0.92*	−0.22	0.87**	−0.61	−0.85**
ACT-L	−0.22	−0.29	−0.22	−0.59	−0.05	0.41	0.19	−0.37	−0.38
PAS-B	0.66	0.56	0.62	0.72	0.51	−0.47	−0.36	0.67	0.72
PAS-T	0.70	0.55	0.69	0.74	0.60	−0.45	−0.44	0.65	0.78
PAS-L	0.88**	0.71	0.85**	**0.89**	0.73	−0.52	−0.53	0.55	0.95**
TOT-B	0.31	0.44	0.06	0.38	−0.09	−0.68	0.16	0.37	0.26
TOT-T	0.65	0.54	0.56	0.66	0.47	−0.55	−0.29	0.59	0.68
TOT-L	0.86**	0.63	0.87**	0.80	0.81	−0.40	−0.56	0.62	0.92**
GRO-B	−0.32	−0.28	−0.57	−0.65	−0.44	−0.24	0.55	−0.52	−0.52
GRO-T	0.51	0.44	0.35	0.23	0.30	−0.37	−0.05	−0.25	0.37
GRO-L	0.62	0.53	0.52	0.42	0.43	−0.31	−0.20	−0.12	0.53
DIG-B	0.42	0.09	0.68	0.49	0.72	0.05	−0.74	−0.16	0.63
DIG-T	−0.19	−0.53	0.25	−0.03	0.42	0.62	−0.65	−0.37	0.09
DIG-L	−0.81**	−0.82**	−0.52	−0.69	−0.34	0.89**	0.06	−0.25	−0.70
RER-B	0.19	0.12	0.10	−0.38	0.25	0.43	0.16	0.46	−0.09
RER-T	0.26	0.00	0.50	0.03	0.64	0.65	−0.46	0.34	0.27
RER-L	0.32	0.00	0.67	0.28	0.77	0.59	−0.71	0.29	0.47
3-Ch_T	−0.42	−0.03	−0.79	−0.43	−0.89**	−0.45	0.84**	−0.26	−0.63

Abbreviations: wt (weight), Hippo (hippocampus), CC (corpus callosum), LV (lateral ventricle), 3 V (3^rd^ ventricle), 4 V (4^th^ ventricle), GP (globus pallidus), SNIF- (sniffing of other body parts), ACT- (active contacts), PAS- (passive contacts), TOT- (total social contacts), GRO- (self-grooming), DIG- (digging), RER- (rearings), 3-Ch_T (3-chamber test sociability time).

### *Negr1* deficiency leads to impaired neuritogenesis in hippocampal neurons

**Figure 5:**
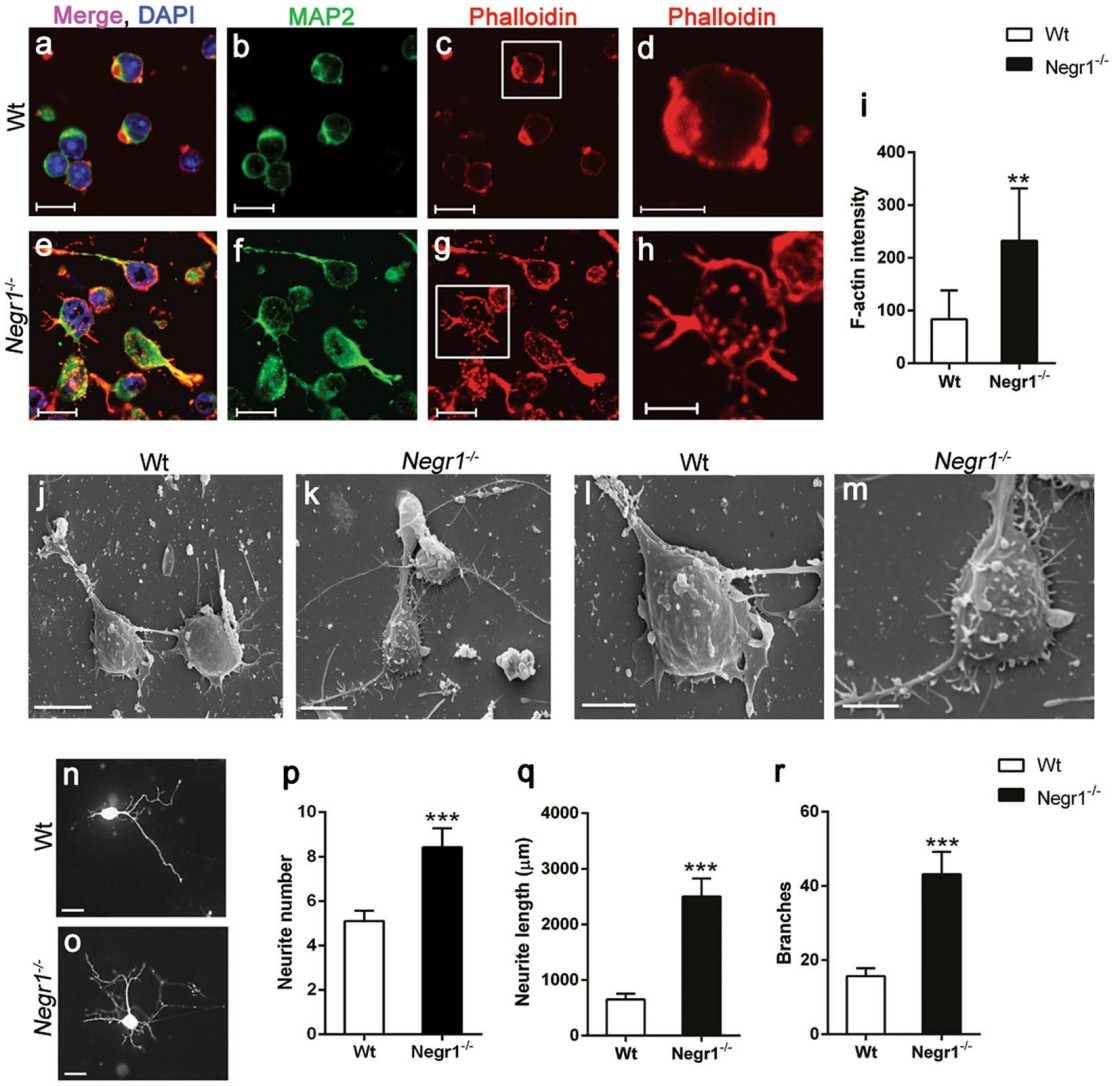
Neuritogenesis is impaired in *Negr1*^*−/−*^ hippocampal neurons. Confocal images of DIV 0.25 hippocampal neurons derived from P0–1 Wt (**a–d**) and *Negr1*^*−/−*^ (**e–h**) mice. Phalloidin staining marks F-actin (red), MAP2 for neurons (*green)*, and DAPI stains nuclei (*blue)*. Scale bar is 10 μm. Graph (**i**) represents quantification of F-actin intensity for actin aggregates at the neurite initiation site (μm^2^). Scanning electron microscopy images of Wt (**j,l**), *Negr1*^*−/−*^ (**k,m**), hippocampal neurons at DIV0.25. Scale bar in (**j**,**k**) is 5 μm, in (**l**,**m**) 2 μm. Representative images of pAAV-hSyn-RFP transfected hippocampal neurons derived from Wt (**n**) and *Negr1*^*−/−*^ (**o**) mice. Scale bar is 50 μm. Graph (**p–r**) represents the number, length, and branch points of the neurites per neuron obtained by neuron tracing Neuroleucida. N = 75–82 neurons evaluated per genotype from three independent experiments. Data represent mean ± SEM, *p < 0.05, **p < 0.01, ***p < 0.001, Mann–Whitney *U* test (Wilcoxon rank sum test).

To examine whether *Negr1* is involved in the initiation stage of the formation of neurites and their outgrowth, we prepared dissociated hippocampal neuronal culture from postnatal (P) day P0–1 of *Negr1*^*−/−*^ mice and the corresponding Wt mice. The cytoskeleton of neurite sprouting was examined at 6 hrs post plating with immunolabelling and scanning electron microscopic imaging. F-actin binding compound phalloidin was used to label growing actin filament aggregates through the spherical neuronal cells at the neurite growth initiation site^42^, and the neuronal marker MAP2 that labels microtubules of the neurons was used to distinguish neurons from glia. We observed that spherical Wt neurons started to develop lamellopodia with few filopodia protrusions (Fig. 5a–d). In contrast, the *Negr1*^*−/−*^ neurons possess large F-actin rich protrutions that began to aggregate with diffused lamellopodia and a higher number of filopodia that develop faster compared to control neurons (Fig. 5e–h). Quantification of F-actin intensity revealed a significant (p < 0.001) increase in neurite initiation sites in *Negr1*^*−/−*^ hippocampal neurons (Fig. 5i). Similar topographical features of profound filopodia protrusions on the surface and accelerated neurite sprouting in *Negr1*^*−/−*^ neurons were also visualised by scanning electron microscopic images (Fig. 5j–m). Tracing of neuronal development was also done by transfecting the hippocampal culture at DIV2 with plasmid expressing RFP only in neurons under the synapsin promoter. Morphometric analysis of neurite outgrowth and branching in transfected neurons was examined 24 hrs after transfection (at DIV3). Our analysis showed that *Negr1* deficiency led to a significant (p < 0.0001) increase in neurite number, neurite length and branching at DIV3 (Fig. 5n–r; Supplementary Table S3).

## Discussion

The present study expands the phenotyping of *Negr1*^*−/−*^ mice and sheds light on the relationship between observed neuroanatomical and behavioral patterns. We show that *Negr1*^*−/−*^ mice possess neuroanatomical and behavioral endophenotypes which are related to the core diagnostic domains of several psychiatric disorders like SCZ, ASD and ADHD. NEGR1 has been implicated in normal brain development and susceptibility to a wide spectrum of psychiatric disorders and in AD pathology in humans. Therefore, it is important to ask the question of how the alterations of NEGR1 expression may underlie the neuropathology of psychiatric disorders.

*Negr1* expression was observed in cortical-subcortical brain areas that are known to be important for cognitive, affective and motivational behavior. Importantly, we observed intensive expression of *Negr1* in thalamic reticular nucleus which is the functional hub for information flow in thalamo-cortical circuits. High *Negr1* expression was also detected in the islands of Calleja which modulate dopamine signalling between PFC and temporal lobe, in the ventral striatopallidal system. These nuclei play a significant role in maintaining normal connectivity of brain and their alterations are related to the pathophysiology of psychiatric disorders^43,44^.

MRI-based volumetric analysis revealed neuroanatomical abnormalities in *Negr1*^*−/−*^ mice, including an enlargement of ventricles, and a decrease in the volume of the total brain, hippocampus, globus pallidus, and corpus callosum. These anomalies corroborate reports of animal models of several psychiatric disorders summarised in Supplementary Table S4. Smaller total brain and hippocampal volume in *Negr1*^*−/−*^ mice is in line with our previous study showing impaired learning and sociability in *Negr1*^*−/−*^ mice^40^. The hippocampus is essential for the formation of memory, spatial navigation, learning, emotional and social behavior through its widespread connections with the PFC, amygdala, thalamus, hypothalamus and basal ganglia^45^.

Reduced total brain and hippocampal volume has been observed in several animal models, such as a SCZ model^46^, a MDD model^47^, ASD model^48^, and a post-traumatic stress disorder (PTSD) model^49^. Smaller globus pallidus in *Negr1*^*−/−*^ mice is indicative of disrupted cortico-basal ganglia circuitry and/or connections of the limbic pallidum with the dopaminergic system^50^. The globus pallidus and striatum are components of the basal ganglia that make connections with the PFC and thalamus. In addition, they are involved in the reward prediction, memory, attention and movement planning^51,52^. Several studies indicate that the globus pallidus is also related to the pathophysiology of depressive disorders^53^, Tourette syndrome, obsessive compulsive disorder, ADHD and accompanying neuropsychiatric symptoms associated with PD and Huntington’s disease^50^. A reduction in globus pallidus size has also been observed in ITGβ3 model of ASD^48^. These results corroborate that the reductions in the volume of brain areas present in *Negr1*^*−/−*^ mice are similar to brain endophenotypes of several neuropsychiatric disorders.

Enlarged ventricles observed in *Negr1*^*−/−*^ mice have been correlated with the negative symptoms of schizophrenia^54^, psychotic behavior in depression^55^, and autistic behavior^56^. Enlargement of ventricles has also been observed in SCZ animal models like hDISC, *Zic2*^*kd/*+^, *Df16A*+/−, 22q11.2, CRMP2, NCAM180^48,57–60^, in an ASD model like 15q13.3^61^, and in SrGAP3 animal model of mental retardation^62^. These findings support the hypothesis of the genetic involvement of *Negr1* in the pathogenesis of psychiatric disorders.

Reduced corpus callosum volume observed in *Negr1*^*−/−*^ mice is in line with a study^23^ which showed that specific variants of the *NEGR1* gene were linked to lower white matter integrity of the corpus callosum and fornix. A similar phenotype has also been found in several mouse models of SCZ such as MAP6 KO^63^, DISC1_tr_^64^, *Zic2*^*kd/*+^ ^59^, in an ASD mouse model ITGβ3^48^ and in an animal model of MDD^65,66^. Corpus callosum is the largest white matter tract mediating information flow between two cerebral hemispheres via excitatory and inhibitory neurotransmission^67^. The hypothesis of imbalance in the ratio between excitation (E) and inhibition (I), called the E/I balance due to reduced corpus callosum volume has been suspected in psychiatric disorders like autism, SCZ and other overlapping phenotypes of mental disorders^68^. Structural changes in corpus callosum have also been linked with faulty hemispheric connectivity and are associated with impaired sensory motor, social, emotional and cognitive functions^69,70^. Furthermore, Negr1^*−/−*^ mice were found to exhibit increased susceptibility to pentylenetetrazol (PTZ)-induced seizures which may reflect E/I imbalance^40^. Therefore, we hypothesize that social impairments in *Negr1*^*−/−*^ mice might be influenced by E/I imbalance due to reduced corpus callosum volume.

Immunohistochemical analysis of hippocampus revealed significant reduction in PV positive interneurons with unchanged number of NeuN positive nuclei in *Negr1*^*−/−*^ mice as compared to Wt mice. Recent study showed significant decrease in adult hippocampal neurogenesis in the *Negr1*^*−/−*^ mice with no change in NeuN positive neurons and hippocampal neurites^71^. Our previous study describes the impaired entorhinal axonal growth and abnormal entorhinal fibre projections in the hippocampus of *Negr1*^*−/−*^ mice^40^. Defective neuronal migration in the somatosensory cortex and decrease in spine density were also reported in *Negr1*^*−/−*^ mice^41^. Taken together we suggest that Negr1 deficiency in *Negr1*^*−/−*^ mice does not affect the total number of neurons rather it is interchanging some specific subtypes of neurons or newly born neurons.

Decrease in GABAergic signalling is among the most likely pathophysiological mechanisms causing psychiatric disorders like SCZ, ASD, MDD, stress and anxiety^72–76^. Increasing evidence suggest that a decrease in the activity of parvalbumin-expressing inhibitory interneurons is due to the reduced excitability of neurons^75,77^. A recent study showed decreased long term potentiation (LTP) and miniature excitatory postsynaptic currents (EPSCs) in the hippocampus of *Negr1*^−/−^ mice^71^. Profound alterations in the distribution of functional neurotransmitter receptors in the hippocampus has been also shown in *Negr1*^−/−^ mice^40^. Therefore, the reduced number of PV positive interneurons could influence overall hippocampal activity by decreasing inhibitory signals, possibly leading to hyperactivated hippocampus. Alternatively, it could be a compensatory mechanism for reductions of excitatory tone due to reduced corpus callosum volume. Altogether, we suggest that Negr1 deficiency does not directly influence the number of neurons, comparatively it plays an important role in neuronal migration, axonal projection and circuit formation. Additionally, Negr1 is also essential for balancing the E/I ratio for proper synaptic transmission.

Abnormal social behavior is a common feature of several psychiatric disorders. Decline of social interactions and social withdrawal that is one of the negative symptoms of SCZ. One important feature of *Negr1*^*−/−*^ mice observed during this study was the lack of barbering behavior or whisker trimming, a behavior commonly seen in the Wt mice in our animal facility, which reflects cooperative social activity and cognition and is evident in several group-housed mouse strains^78,79^. Whisker trimming or barbering, associated with dominance, is commonly observed in the C57BL/6 strain as an index of social hierarchy^80,81^. Lack of whisker trimming has also been described as an indication of inability to establish social hierarchy and impaired social cognition^82^. Interestingly, a drastic decrease in barbering behavior was also present in mice with the deletional mutation of another IgLON family gene, *Lsamp*^83^. From the previous study, we know that the social stimuli are less attractive for *Negr1*^*−/−*^ mice. In the 3-chamber test, Wt mice clearly preferred the presence of a conspecific, compared to an empty room, whereas in their *Negr1*^*−/−*^ littermates no such effect was seen^40^. In the current study, we evaluated the aspects of social interaction of *Negr1*^*−/−*^ mice in more detail. We show that the lack of preference of social stimuli in the 3-chamber test could be due to deficits in social memory/recognition, as *Negr1*^*−/−*^ mice spent equal amount of time in all three chambers, showing no preference for the rooms with either familiar or unknown conspecific (Supplementary Fig. S5). However, in our current test, the common preference of rodents to investigate a novel conspecific more than a familiar one was only evident as a tendency in Wt mice, therefore the possible deficits in social recognition in *Negr1*^*−/−*^ mice need to be clarified in future studies.

During the DSI test, the total time spent in social contact with an unknown partner was not different in *Negr1*^*−/−*^ mice compared to their Wt littermates. However, *Negr1*^*−/−*^ mice made more approaches towards their partner compared with Wt mice whereas the duration of each interaction bout was relatively shorter, indicating disoriented and confused social behavior of *Negr1*^*−/−*^ mice. We have described similar behavioral pattern of *Negr1*^*−/−*^ mice in the tube dominance test^40^ where the winning time of *Negr1*^*−/−*^ mice was significantly shorter. All together, these results indicate that *Negr1*^*−/−*^ mice display hyperactivity in the social contacts engaging only in brief superficial bouts of social contact. Moreover, *Negr1*^*−/−*^ mice show reduced self-grooming, a measure of repetitive behavior that are highly stereotyped patterns of sequential movements^84^. The reduced self-grooming in *Negr1*^*−/−*^ mice could also be caused by increased digging and rearing time, exhibiting hyperactivity and behavioral perseverations. Decreased self-grooming has been also observed in mice lacking D1A dopamine receptors^85^, and in 16p11^+/−^ mice which is associated with ASD and other neurodevelopmental disorders^86^. We also performed the marble burying test, reflecting obsessive-compulsive behavior^87^. *Negr1*^*−/−*^ mice showed no difference in marble burying as compared with controls despite having a higher number of digging bouts in the DSI test. This indicates that in *Negr1*^*−/−*^ mice digging in the DSI test does not reflect repetitive obsessive-compulsive behavior, but is rather an effort to escape or find a shelter^88^.

*Negr1*^*−/−*^ mice performed less supported rearings in the RI and OF tests, but more supported rearings in the DSI test, showing that this behavioral parameter is heavily dependent on the test type. Similar to the DSI test, neither *Negr1*^*−/−*^ nor Wt mice displayed aggressive or attacking behavior in the RI test. It is possible that the absence of aggression in these animals is influenced by the mixed (129S5/SvEvBrd × C57BL/6) genetic background of the mice. In fact, reduced aggression level probably enables us to observe more subtle aspects of social behavior^89^.

In the present study, we draw correlations between brain structure and behavioral parameters. MRI imaging was done on the same set of mice used for the behavioral testing. Volumetric alterations in the ventricles, hippocampus, globus pallidus, and corpus callosum in *Negr1*^*−/−*^ mice were most significantly correlated with activities like sniffing bouts of other body parts, and the duration of active and passive contact during social interactions. Although the obtained correlative data need to be taken with caution since the number of mice used for the MRI experiment was limited, our findings provide initial evidence that the rate of alterations in the brain structures could be correlated with the behavioral changes present in the *Negr1*^*−/−*^ mice.

To validate the putative essential role of Negr1 in the brain development by regulating neuronal outgrowth and synapse formation, we investigated the early neurite sproutings in the developing hippocampal neurons derived from *Negr1*^*−/−*^mice. Compared to later stages of neuronal development and function (dendritic and axonal development), the role of Negr1 in the early steps of neuritogenesis has not been well described. Proteins regulating actin, such as F-actin, mark the initial sprouting of the neurites at the neurite initiation site^90^. Here, we show for the first time defective F-actin accumulation and filopodia at neurite initiation stage of neuritogenesis in *Negr1*^*−/−*^ hippocampal neurons. Enhanced neurite outgrowth was also recorded at DIV3, indicating disruption in the initial dynamics of cytoskeleton formation during neuritogenesis. Previous reports show that downregulation of NEGR1 through siRNA approach impairs/lowers neuronal arborisation^39,91^. The possible explanation for this contrasting result could be the difference in time points and a different source of neuronal cells. Despite the incongruence in the results it is evident that Negr1 is a central factor regulating neuronal morphology at different developmental stages during neurite formation. IgLONs are known to form dimers through homophilic and heterophilic interactions and control neuronal growth and synaptogenesis^38,91–94^. For example, it has been shown that Ntm mediates bifunctional effects on neurite outgrowth through attractive and repulsive mechanisms, which are cell type specific^95^. The combined effect of Lsamp and Ntm regulates neuritogenesis through complementary interaction, which was independent of their cell-cell adhesion functions^96^. Neuritogenesis involves multiple interactions between the developing neurites and the extracellular matrix. Constructural changes during neuritogenesis were related to abnormal neural circuit development in ASD and SCZ^97,98^. Taken together, our results indicate that Negr1 regulates the structural molecules of neurite initiation stage during neuritogenesis even before any connections with other neighbouring neurons are made.

In conclusion, the present study demonstrates the importance of Negr1 at brain structure and function level. We show that *Negr1* deficient mice exhibit behavioral alterations and morphological abnormalities in the brain that are similar to the ones observed in psychiatric disorders such as SCZ, ASD and ADHD. The link between neuroanatomical and behavioral findings is significant for understanding the neuronal development and structural changes underlying neuronal connectivity problems related to the etiology of psychiatric disorders. Further research is required to elaborate the structural and functional connectivity of the neural network in this mouse model. This knockout mouse line can be a useful model to elucidate the key molecular targets for the development of new therapeutic strategies in neuropsychiatric research.

## Methods

### Animals

Male wild-type [Wt] mice and their homozygous *Negr1-*deficient littermates [*Negr1*^*−/−*^], described previously^99^ in F2 background [(129S5/SvEvBrd × C57BL/6) × (129S5/SvEvBrd × C57BL/6)] were used in the present study. Additional Wt and *Negr1*^*−/−*^ mice pups were generated from a separate breeding pair on a similar background to perform primary culture experiments. Mice were group-housed in standard laboratory cages measuring 42.5 (L) × 26.6 (W) × 15.5 (H) cm, 6–8 animals per cage in the animal colony at 22 ± 1 °C, under a 12:12 h light/dark cycle (lights off at 19:00 h). A 2 cm layer of aspen bedding (Tapvei, Estonia) and 0.5 l of aspen nesting material (Tapvei, Estonia) were used in each cage and changed every week. Water and food pellets (R70, Lactamin AB, Sweden) were available *ad libitum*. Breeding and the maintenance of the *Negr1*^*−/−*^ mice were performed at the animal facility of the Institute of Biomedicine and Translational Medicine, University of Tartu, Estonia. The use of mice was conducted in accordance to the regulations and guidelines approved by the Laboratory Animal Centre at the Institute of Biomedicine and Translational Medicine, University of Tartu, Estonia. All animal procedures were conducted in accordance with the European Communities Directive (2010/63/EU) with permit (No. 29, April 28, 2014) from the Estonian National Board of Animal Experiments.

### *In situ* hybridisation and neurofilament immunostaining

*Negr1* (650 bp) transcripts were cloned from a cDNA pool of a C57BL/6 mouse brain and inserted into pBluescript KS vector (Stratagene, La Jolla, CA) to create an *in situ* probe. RNA *in situ* hybridization on sagittal and coronal free-floating PFA-fixed 40 μm mouse brain cryosections using digoxigenin-UTP (Roche) labelled *Negr1* antisense RNA probes was performed as described previously^100^. Neurofilament immunohistochemistry was carried out with mouse anti-2H3 antibody, (1:100; Developmental Studies Hybridoma Bank) following the peroxidase method as described previously^96^. Images were captured using inverted light microscope (Olympus BX61 microscope) equipped with Olympus DX70 CCD camera (Olympus, Hamburg, Germany).

### Magnetic resonance imaging

At 7 months of age, mice were deeply anesthetized and perfused with 0.1 M PBS followed by 4% paraformaldehyde (4 °C). Brains were left in the skulls to preserve the anatomy and incubated in 4% PFA at 4 °C and then in PBS until 2 days prior to imaging. Skulls were then placed in 2 mM gadovist in PBS and incubated at 4 °C with rocking until imaging. A T2 RARE sequence was used for imaging using a 94/20 Bruker BioSpec small animal MRI with the following parameters: Tr, 900 ms; TE, 47.13 ms; imaging matrix, 512 × 360 × 80; spatial resolution, 0.0444 × 0.03 × 0.2 mm for an imaging time of approximately 3 h and 4 min. Volumes were segmented manually by an observer blinded to the genotype using ITK-SNAP (V3.2.0). The entire brain and the ventricles were manually delineated^101^ for each slice and their 3D volumes were measured. A total of 13 mice were tested (Wt: n = 6; *Negr1*^*−/−*^: n = 7).

### Immunohistochemistry analysis of hippocampus

Fluorescent immunohistochemistry was performed on floating 40 µm thick sagittal sections collected after every 240 μm to 1X PBS. Sections were permeabilized, blocked and immunostained with mouse anti-NeuN antibody (1:250, Millipore; MAB377) in combinations with guinea pig anti-Parvalbumin (PV) antibody (1:200, Synaptic Systems; 195 004) followed by secondary antibody (FITC AffiniPure donkey anti gunie-pig (1:1000, Jackson ImmunoResearch Lab., 706-095-148, TRITC AffiniPure donkey anti-mouse (1:1000, Jackson ImmunoResearch Lab., 715-025-150) and DAPI. Subsequently sections were rinsed with PBS and mounted with Fluoromount mounting medium (Sigma Aldrich), and covered with a 0.17-mm coverslip (Deltalab). NeuN-positive nuclei, and Parvalbumin-positive cell counting was performed with every 6^th^ section and quantified as the number of NeuN-positive or PV positive cells/area mm^2^ using ImageJ Software (version 1.52a; National Institutes of Health).

### Behavioral testing

Behavioral testing was performed between 9:00 A.M. to 17:00 P.M. under 30 lux light intensity. Behavioral testing started when the mice were 12–14 weeks old and the same mice were repeatedly used in the behavioral tests. The testing order was as follows: three –chamber test, direct social interaction, marble burying and tail-suspension. Resident intruder test and Open field test was performed with different set of mice. Before each experiment, mice were let to habituate to the experimental room and the lighting conditions therein for 1 h. The mice were allowed to rest 1–2 weeks between the tests.

### Barbering behavior

Barbering behavior was estimated in group-housed male mice (7–9 animals per cage) on the following three- scale: (1) no whiskers, (2) partially trimmed whiskers and (3) full whiskers, and at two time-points (8–9 and 20–21 weeks of age). The percentage of mice having full, partially trimmed and no whiskers was calculated. A total of 67 mice were observed (Wt: n = 33; *Negr1*^*−/−*^: n = 34).

### Direct social interaction test

The social interaction test was carried out as described previously^83^, briefly: 10 pairs of two unfamiliar mice of same genotype were matched according to the body weight and their behavior was video-recorded for 10 min. The videos were later scored by a trained observer. Three measures (time spent in s, the number of bouts, and average bout duration) were registered for each mouse for the following parameters: (1) sniffing the body of the other mouse, (2) anogenital sniffing of the other mouse, (3) passive contact, (4) rearing, (5) digging, (6) aggressive attack, and (7)self-grooming. Parameters 1, 2 and 3 were also summarized to get an additional parameter of “total social contact time” for each animal. Altogether, 40 mice were tested (Wt: n = 20; *Negr1*^*−/−*^: n = 20).

### Marble burying test

Twenty glass marbles (1.5 cm in diameter) were placed on 5 cm of sawdust bedding as a 4 × 5 grid in a Plexiglas cage measuring 42.5 (L) × 26.6 (W) × 15.5 (H) cm. The mice were placed in the box individually for 30 min, and the number of marbles buried at least two-thirds deep were counted. A total of 40 mice were tested (Wt: n = 20; *Negr1*^*−/−*^: n = 20).

### Tail suspension test

Mice were suspended for 6 min from the edge of a shelf 58 cm above a table top by adhesive tape, placed approximately 1 cm from the tip of the tail. The duration of immobility was scored during the last 4 min from the recorded videos by an observer blind to the genotype. Mice were considered immobile only when they hung passively and completely motionless for at least 3 seconds. A total of 32 mice were tested (Wt: n = 16; *Negr1*^*−/−*^: n = 16).

### Resident-intruder test

Previously group-housed males were separated and housed individually for 1–2 months before testing. A group-housed mouse of the same age and same genotype was used as an intruder mouse. The resident animal was placed from its home cage into a separate small cage and left alone for 30 minutes. After 30 minutes an intruder was introduced into the same cage. The test was stopped immediately after the first attack (an attack being defined as a bite) and lasted up to 5 min if no attack occurred. The interactions between the two animals were videotaped for 5 minutes from above and later scored for further analysis. The number of animals engaged in aggressive and non-aggressive social behavior (sniffing, anogenital sniffing rearings, digging and self-grooming) was recorded allowing the comparison of three parameters (time spent in s, the number of bouts, and average bout duration). Altogether, 40 mice were tested (Wt: n = 20; *Negr1*^*−/−*^: n = 20).

### Open field test

Open field test were performed as reported earlier^96^. Altogether, 60 mice were tested (Wt: n = 30; *Negr1*^*−/−*^: n = 30).

### Primary hippocampal culture and assessment of early neuritogenesis

Assessments of early neuritogenesis were performed as reported earlier^96^. To study the neurite initiation stage, 6 hr post culturing neurons were prepared for scanning electron microscopy; immunostaining and quantification of F-actin were done. For neurite outgrowth and branching analysis, pAAV-hSyn-RFP transfected primary hippocampal neurons were imaged on DIV3 and neurite number, neurite length and branches were analysed as reported earlier^96^.

### Statistical analysis

Data were analysed using Statistica V12 (Statsoft Inc., Oklahoma, USA) and graphs were plotted using Prism 5 (GraphPad, La Jolla, CA, USA). Differences in continuous variables were evaluated by unpaired *t*-test, one-way ANOVA or repeated measures ANOVA followed by Mann-Whitney *U* test as a *post hoc* test (Wilcoxon Rank Sum test) or two-way ANOVA followed by Newman-Keuls *post hoc* test. Chi-square one-sample analysis was used to analyse the results of whisker trimming. As the behavioral scores were not normally distributed, Spearman’s rank-order method was used for the calculation of correlation coefficients. The differences were considered to be significant if the p-values were less than 0.05. All results are displayed as mean ± SEM (standard error of mean).

### Ethical approval

All animal procedures in this study were performed in accordance with the European Communities Directive (2010/63/EU) and permit (No. 29, April 28, 2014) from the Estonian National Board of Animal Experiments. In addition, the use of mice was conducted in accordance to the regulations and guidelines approved by the Laboratory Animal Centre at the Institute of Biomedicine and Translational Medicine.

This article does not contain any studies with human participants or human samples.

## Supplementary information

Supplementary dataset1

## Data Availability

The datasets generated during and/or analysed during the current study are available from the corresponding author on reasonable request.
