# Supplementary material for: Neural cell adhesion molecule Negr1 deficiency in mouse results in structural brain endophenotypes and behavioral deviations related to psychiatric disorders
Source: Sci Rep. 2019 Apr 1;9:5457. doi: 10.1038/s41598-019-41991-8 (PMC6443666; doi:10.1038/s41598-019-41991-8)

Supplementary materials

Neural cell adhesion molecule *Negr1* deficiency in mouse results in structural brain endophenotypes and behavioral deviations related to psychiatric disorders

Katyayani Singh [a,b] *, Mohan Jayaram [a,b], Maria Kaare [a,b], Este Leidmaa [c], Toomas Jagomäe [a,b], Indrek Heinla [d], Miriam A. Hickey [e], Allen Kaasik[e], Michael K. Schäfer[f], Jürgen Innos [a,b], Kersti Lilleväli [a,b], Mari-Anne Philips [a,b], Eero Vasar [a,b]

[a] *Department of Physiology, Institute of Biomedicine and Translational Medicine, University of Tartu, 19 Ravila Street, 50411 Tartu, Estonia*

[b] *Centre of Excellence in Genomics and Translational Medicine, University of Tartu, 19 Ravila Street, 50411 Tartu, Estonia*

[c] *Institute of Molecular Psychiatry, University of Bonn Sigmund-Freud-Str.25 53125 Bonn, Germany.*

[d] *Department of Psychology, UiT The Arctic University of Norway, Postboks 6050 Langnes, 9037 Tromsø, Norway*

[e] *Department of Pharmacology, Institute of Biomedicine and Translational Medicine, University of Tartu, 19 Ravila Street, 50411, Tartu, Estonia*

[f] *Department for Anesthesiology, University Medical Center and Focus Program Translational Neuroscience (FTN), Johannes Gutenberg-University Mainz, Mainz, Germany.*

*** Correspondence:** singhkat@ut.ee, katyayani.micro@gmail.com
https://orcid.org/0000-0002-3287-1885

Figure S1: Neuronal growth regulator (NEGR1) mRNA expression in the adult brain. In situ hybridisation for Negr1 antisense probe in the Wt (Negr1+/+) **(a)** and Negr1-/- **(b)** coronal brain section. Negr1-/- brain section is devoid of any labelling by Negr1 antisense probes. Scale bars: 1mm.

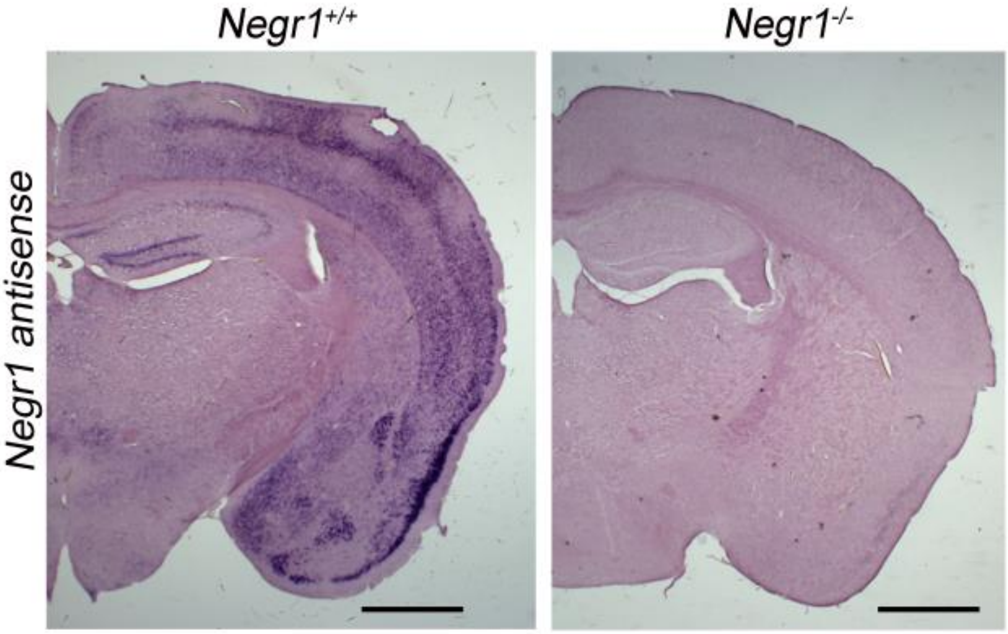

Table S1: Correlations between the MRI indices and social indices of interest in Wt mice. The behavioral measures have been presented in either bouts (-B), time (-T) and bout length (-L), bold numbers represent significant differences, * $p<0.05$ (Spearman's rank-order correlation). Abbreviations: wt (weight), Hippo (hippocampus), CC (corpus callosum), LV (lateral ventricle), 3V (3rd ventricle), 4V (4th ventricle), GP (globus pallidus), SNIF- (sniffing of other body parts), ACT- (active contacts), PAS- (passive contacts), TOT- (total social contacts), GRO- (self-grooming), DIG- (digging), RER- (rearings), 3-Ch_T (3-chamber test sociability time).

	Body wt	Brain wt	Total Brain	Hippo	CC	LV	3V	4V	GP
**SNIF-B**	0.52	0.44	0.23	0.05	0.52	0.56	0.77	0.49	-0.02
**SNIF-T**	0.18	-0.79	0.56	0.47	0.29	0.13	0.36	0.71	0.26
**SNIF-L**	-0.19	**-0.94***	0.25	0.31	-0.09	-0.19	-0.13	0.28	0.17
**ACT-B**	0.51	0.27	0.31	0.08	0.48	0.53	0.79	0.62	-0.01
**ACT-T**	0.18	-0.77	0.59	0.43	0.28	0.17	0.39	0.78	0.26
**ACT-L**	-0.19	**-0.94***	0.31	0.35	-0.04	-0.16	-0.15	0.32	0.24
**PAS-B**	-0.27	0.44	0.19	0.07	0.38	0.42	-0.58	-0.00	0.70
**PAS-T**	-0.76	-0.09	-0.41	-0.38	-0.06	0.51	-0.57	-0.08	0.24
**PAS-L**	-0.78	-0.12	-0.43	-0.41	-0.11	0.49	-0.57	-0.08	0.22
**TOT-B**	0.23	0.49	0.39	0.12	0.66	0.74	0.27	0.53	0.48
**TOT-T**	-0.76	-0.12	-0.39	-0.36	-0.05	0.52	-0.56	-0.05	0.26
**TOT-L**	-0.77	-0.15	-0.41	-0.37	-0.09	0.48	-0.58	-0.08	0.23
**GRO-B**	0.58	0.55	-0.21	-0.12	0.27	0.25	**0.87***	0.01	-0.49
**GRO-T**	0.66	0.77	-0.18	-0.06	0.14	-0.11	0.65	-0.29	-0.51
**GRO-L**	0.60	0.64	0.13	0.22	0.02	-0.57	0.15	-0.44	-0.19
**DIG-B**	0.63	0.03	**0.85***	**0.85***	0.47	-0.43	0.02	0.12	0.52
**DIG-T**	0.04	0.25	-0.12	-0.24	-0.58	-0.64	-0.17	-0.37	-0.37
**DIG-L**	-0.12	0.24	-0.38	-0.52	-0.75	-0.52	-0.13	-0.39	-0.58
**RER-B**	**0.91***	0.26	0.26	0.38	0.31	-0.25	**0.87***	0.07	-0.35
**RER-T**	0.68	0.31	0.33	0.16	0.42	0.27	**0.86***	0.47	-0.13
**RER-L**	-0.49	-0.05	0.06	-0.34	0.20	**0.93***	-0.09	0.67	0.42
**3-Ch_T**	-0.25	0.03	-0.02	-0.39	-0.61	-0.29	-0.29	0.01	-0.16

Table S2: Correlations between the MRI indices and social indices of interest in both genotypes tested. The behavioral measures have been presented in either bouts (-B), time (-T) and bout length (-L), bold numbers represent significant differences, * p<0.05, ** p<0.01, ***p<0.001 (Spearman's rank-order correlation). Abbreviations: wt (weight), Hippo (hippocampus), CC (corpus callosum), LV (lateral ventricle), 3V (3rd ventricle), 4V (4th ventricle), GP (globus pallidus), SNIF- (sniffing of other body parts), ACT- (active contacts), PAS- (passive contacts), TOT- (total social contacts), GRO- (self-grooming), DIG- (digging), RER- (rearings), 3-Ch_T (3-chamber test sociability time)

	**Body wt**	**Brain wt**	**Total Brain**	**Hippo**	**CC**	**LV**	**3V**	**4V**	**GP**
**SNIF-B**	-0.04	0.15	-0.23	-0.11	-0.25	-0.03	**0.58***	-0.11	-0.23
**SNIF-T**	-0.07	-0.44	-0.01	0.02	-0.17	-0.06	0.49	0.16	-0.08
**SNIF-L**	-0.01	**-0.60***	0.23	0.12	0.13	0.04	-0.11	0.29	0.14
**ACT-B**	-0.22	-0.03	-0.42	-0.34	-0.48	0.16	**0.75***	-0.02	-0.43
**ACT-T**	-0.37	-0.51	-0.39	-0.35	-0.55	0.17	**0.74***	0.01	-0.40
**ACT-L**	-0.18	**-0.75***	0.09	0.03	-0.06	0.05	0.05	0.09	0.04
**PAS-B**	0.26	0.40	0.19	0.14	0.19	0.06	-0.17	0.54	0.30
**PAS-T**	-0.23	0.05	0.03	0.09	0.22	-0.08	-0.36	-0.04	0.39
**PAS-L**	-0.30	-0.02	0.00	0.07	0.18	-0.11	-0.34	-0.16	0.35
**TOT-B**	0.11	0.37	-0.06	-0.06	-0.09	0.15	0.31	0.49	0.05
**TOT-T**	-0.26	0.02	0.01	0.07	0.19	-0.07	-0.32	-0.04	0.37
**TOT-L**	-0.29	-0.03	0.02	0.08	0.19	-0.10	-0.35	-0.13	0.37
**GRO-B**	0.47	0.39	0.13	0.23	0.35	-0.33	0.00	-0.28	-0.02
**GRO-T**	**0.63***	0.55	0.38	0.49	0.50	**-0.61***	-0.27	-0.47	0.22
**GRO-L**	**0.66***	0.49	**0.59***	**0.65***	0.55	**-0.76****	-0.44	-0.52	0.46
**DIG-B**	0.00	-0.03	-0.08	-0.27	-0.11	0.57	0.56	0.30	-0.17
**DIG-T**	-0.01	0.01	0.04	-0.07	-0.08	-0.11	-0.31	-0.32	-0.14
**DIG-L**	0.08	0.14	0.12	0.14	0.02	-0.55	-0.33	-0.46	0.00
**RER-B**	**0.65***	0.23	0.37	0.33	0.43	-0.29	0.02	0.02	0.05
**RER-T**	-0.04	-0.03	-0.13	-0.41	-0.03	**-0.72****	0.09	0.51	-0.28
**RER-L**	-0.19	-0.08	-0.25	-0.50	-0.17	**-0.83*****	0.09	0.53	-0.32
**3-Ch_T**	-0.23	0.01	-0.27	-0.11	-0.48	-0.37	0.46	-0.25	-0.19

Table S3: Neuritogenesis measurements for Wt and *Negr1*$^{-/-}$ mice. Data are shown as mean±SEM, DIV=days *in vitro*, μm^2= square micrometers.

**Neuritogenesis**	**Wt**	***Negr1*$^{-/-}$**
**F-actin accumulation**, μm^2 (DIV0.25), n=30+30	82 ± 295	232.2 ± 118
**Neurite number per neuron** (DIV3), n=91+73	5.1 ± 2.2	8.4 ± 3.5
**Neurite length per neuron**, μm (DIV3), n=91+73	652.6 ± 50.99	2504 ± 160.9
**Branch point number per neuron** (DIV3), n=91+73	15.67 ± 10.15	43.17±25.30

Table S4: Comparison between structural MRI findings of *Negr1*$^{-/-}$ mice with different animal model of psychiatric disorders and structural MRI findings in human psychiatric patients. (MDD: major depressive disorder, BP: bipolar disorder, ASD: autism spectrum disorder, SCZ: schizophrenia, ADHD: attention deficit hyperactivity disorder, OCD: obsessive-compulsory disorder, PTSD: post-traumatic stress disorder).

Brain structural anomaly in *Negr1*$^{-/-}$ mice	Psychiatric animal model (ref.)	Psychiatric patients (ref.)
**Enlarged Ventricles**	**MDD:** (Zubenko et al. 2014), WKY rats (Gormley et al. 2016)<br>**SCZ:** ckr (Torres et al. 2005), hDISC1 (Pletnikov et al. 2008; Hikida et al. 2007; Clapcote et al. 2007; Shen et al. 2008), *Zic2*$^{kd/+}$ (Hatayama et al. 2011), *Df16A*+/− , 22q11.2 (Ellegood et al. 2014), CRMP2 (Zhang et al. 2016), NCAM180 (Wood et al. 1998)<br>**Down's syndrome**: Ts1Cje and Ts2Cje (Ishihara et al. 2010)<br>**ASD**: 15q13.3 (Kogan et al. 2015)<br>**Mental retardation**: SrGAP3 (Koschützke et al. 2015)	**MDD:** (Kempton et al. 2011; Schmaal et al. 2017)<br>**BP:** (Swayze et al. 1990; Hibar et al. 2016)<br>**ASD**: (Movsas et al. 2013; Turner et al. 2016)<br>**SCZ:** (Pina-Camacho et al. 2016; van Erp et al. 2016; Del re et al. 2016)<br>**ADHD:** (Wang et al. 2007)<br>**OCD:** (Rosenberg et al. 1993)
**Reduced Hippocampus**	**MDD:** (McIntosh et al. 2017); CYP2C19 (Peerson et al. 2014); WKY rats (Gormley et al 2016)<br>**ASD**: ITGβ3 (Ellegood et al. 2012)<br>**SCZ**: (Johnstone et al. 2011)<br>**PTSD**: (Golub et al. 2011)	**MDD:** (Kempton et al. 2011, Carballedo et al. 2012; MacMaster et al. 2014; Xue et al. 2015, Schmaal et al. 2017; Durmusoglu et al. 2018)<br>**BP:** (MacMaster et al. 2014; Hibar et al. 2016)<br>**ASD**: (Nicolson et al. 2006)<br>**SCZ: (**Shepherd et al. 2015; van Erp et al. 2016; Lieberman et al. 2017)<br>**OCD**: (Kwon et al. 2003)<br>**PTSD**: (O'Doherty et al. 2015)
**Reduced Corpus callosum**	**MDD:** (Kumar et al. 2004; Zubenko et al. 2014; Kieseppä et al. 2010)<br>**SCZ**: MAP6 KO (Gimenez et al. 2017), DISC1 (Shen et al, 2008), *Zic2*$^{kd/+}$ (Hatayama et al. 2011; (Johnstone et al. 2011)<br>**ASD**: ITGβ3 (Ellegood et al. 2012)	**BP:** (Brambila et al. 2004; Kumar et al 2015)<br>**ASD:** (Vidal et al. 2006)<br>**SCZ:** (Kumar et al 2015; Lu et al 2014; Li et al. 2011; Del re et al. 2016)
**Reduced Globus pallidus**	**ASD**: ITGβ3 (Ellegood et al. 2012)	**ADHD**: (Frodl et al. 2012)
**Reduced Frontal cortex**	**SCZ:** *Df16A*+/− , 22q11.2 (Ellegood et al. 2014)<br>**ASD**: ITGβ3 (Ellegood et al. 2012)	**SCZ: (**James et al. 2004; Arnone et al. 2009; Shepherd et al. 2015; Pina-Camacho et al. 2016)<br>**ADHD** (Noordermeer et al. 2017)

Supplementary Fig S5: Result of 3-chamber social novelty test in *Negr1*$^{-/-}$ mice. Time spent in each chamber; n=20+20. Data represent mean ± SEM, Student's t-*test*.

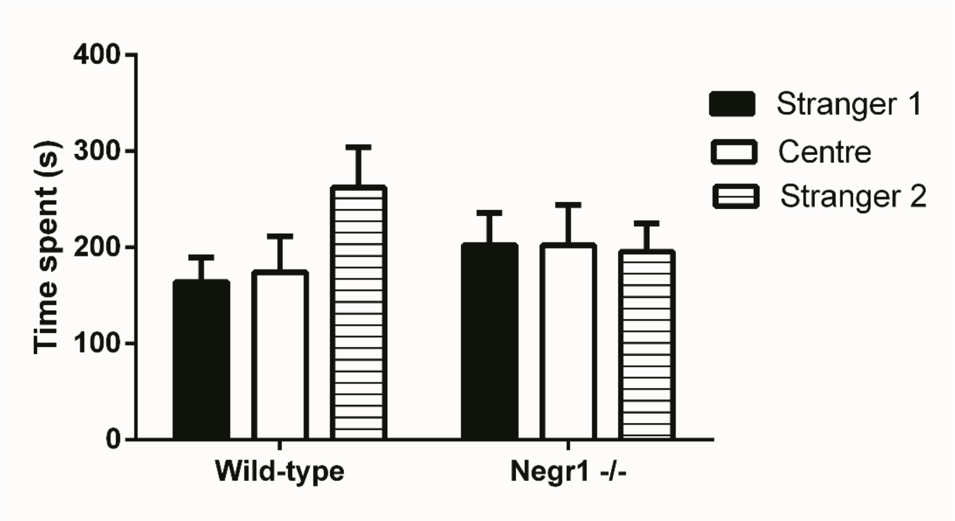

Supplement: Supplementary file 1 — Supplementary dataset1 [file 41598_2019_41991_MOESM1_ESM.pdf]
